# A Balanced Algorithm for In-City Parking Allocation: A Case Study of Al Madinah City

**DOI:** 10.3390/s21093148

**Published:** 2021-05-01

**Authors:** Mohammad A. R. Abdeen, Ibrahim A. Nemer, Tarek R. Sheltami

**Affiliations:** 1 The Faculty of Computers and Information Systems, The Islamic University of Madinah, Al-Madinah 42351, Saudi Arabia; 2 Computer Engineering Department, King Fahd University of Petroleum and Minerals, Dhahran 31261, Saudi Arabia; inemer@kfupm.edu.sa (I.A.N.); tarek@kfupm.edu.sa (T.R.S.)

**Keywords:** balanced parking, multi-objective optimization, Markov chain, optimal routing, Madinah city

## Abstract

Parking in heavily populated areas has been considered one of the main challenges in the transportation systems for the past two decades given the limited parking resources, especially in city centres. Drivers often waste long periods of time hunting for an empty parking spot, which causes congestion and consumes energy during the process. Thus, finding an optimal parking spot depends on several factors such as street traffic congestion, trip distance/time, the availability of a parking spot, the waiting time on the lot gate, and the parking fees. Designing a parking spot allocation algorithm that takes those factors into account is crucial for an efficient and high-availability parking service. We propose a smart routing and parking algorithm to allocate an optimal parking space given the aforementioned limiting factors. This algorithm supports choosing the appropriate travel route and parking lot while considering the real-time street traffic and candidate parking lots. A multi-objective function is introduced, with varying weights of the five factors to produce the optimal parking spot with the least congested route while achieving a balanced utilization for candidate parking lots and a balanced traffic distribution. A queueing model is also developed to investigate the availability rate in candidate parking lots while considering the arrival rate, departure rate, and the lot capacity. To evaluate the performance of the proposed algorithm, simulation scenarios have been performed for different cases of high and low traffic intensity rates. We have tested the algorithm on in-city parking facility in the city of Al Madinah as a case study. The results show that the proposed algorithm is effective in achieving a balanced utilization of the parking lots, reducing traffic congestion rates on all routes to candidate parking lots, and minimizing the driving time to the assigned parking spot. Additionally, the proposed algorithm outperforms the MADM algorithm in terms of the selected three metrics for the five periods.

## 1. Introduction

Traffic congestion on city streets has negative impacts on human life, such as environmental problems, higher energy consumption, limited parking space, psychological damage, noise and air pollution [1]. One of the main causes of this congestion is parking searches due to limited in-city parking space [2] since the resources are limited and building new parking lots is difficult, especially in the city center [3]. Thus, many vehicles on the streets consume more energy and waste their time finding a parking spot in the destination area. According to published research [4,5], it is estimated that around 25–40% of the traffic congestion in the city center is caused by vehicles looking for parking spots and, on average, a driver spends about 7.8 min finding a parking spot [5].

Many algorithms and models have been presented to study the parking problems and reduce the effect of congestion problems, such as PARKAGENT [6], multilayer [7], and CLAMP [8]. Other research studied the prediction of the availability of parking resources based on neural networks [9,10], time series models [11], queueing models [12,13], and multivariate autoregressive models [14]. One of the popular parking systems adopted by traffic authorities in several cities is the parking guidance and, information system (PGIS). The system presents dynamic information about available parking in a form of message board using screens, i.e., variable-message signs (VMS), fitted at main streets and junctions. It can also be broadcast to drivers through the mobile network [15]. This helped reduce travel time for the driver to find an available parking spot [16]. However, the information delivered by PGIS is limited and does not give drivers a full idea about the closest parking facility, its fees, and how a driver is routed to the least congested route to the destined parking spot. Despite the fact that PGIS system increases the probability of finding an available parking spot, it does not provide routing service, given the dynamic changes in traffic conditions and parking availability [17,18]. This might result in imbalanced parking spot distribution and an increase in traffic congestion in some streets and not others. In this research, we propose an intelligent system for selecting and guiding drivers to the suitable parking spot, while considering a set of factors such as walking distance and time to the parking lot, traffic congestion of the streets, the load on the parking gates, parking fees, and the availability of parking resources when the traffic is high or low.

The routing and parking problem considers the parking facilities, the status of the streets, and the drivers’ preferences. Hence, the problem can be represented by a sequence of multiple-objective decision-making points over time, and then the system can allocate the optimal parking spot to drivers based on these objectives. Therefore, the proposed smart routing and parking approach is built to utilize the parking resources in efficient way, minimize traffic congestion on the streets, and equally distribute the vehicles on the parking gates. The availability of the parking spots is implemented based on queueing theory and the dynamic change in the number of spots is designed as a Markov chain. Additionally, all factors are given different weights to study the behaviour of the proposed approach. In this research, we propose a routing and parking algorithm for defining the optimal path to the parking lot based on five factors: traffic congestion rate, waiting time at the parking lot gate, distance to the parking lot, availability in the parking lots, and the cost of the parking spot. We present a queuing model to estimate the availability of a parking spot. We also test and evaluate the algorithm (i.e., availability rate, congestion rate and driving time) for three cases based on the weights of the factors of the multi-objective function under low and high traffic intensity values.

The reminder of this paper is organized as follows. Works related to parking management systems are presented in Section 2. Section 3 introduces the framework of the distributed routing and parking approach. Simulation setup and performance evaluation, based on a case study, are given in Section 4, and the results are discussed in Section 5. Section 6 provides a comparison between our approach and another smart parking algorithm. Finally, Section 7 presents the conclusions and future works.

## 2. Related Works

In this section, the related works are classified into two parking groups: parking reservation group, parking guidance and information group. Then, the most popular algorithms in the parking systems are summarized based on the method used. These works are studied and implemented in the parking problem, so that the driver will find the best parking spot while utilizing the available resources.

### 2.1. Smart Parking Groups

The research into the parking reservation systems and parking guidance and information systems are described in the subsections below.

#### 2.1.1. Parking Reservation Group

Kotb, Shen et al. in [19] presented a new smart parking system based on a set of factors, such as reservation, pricing, and resource allocation. The presented system overcomes some of the parking issues by giving the drivers guaranteed parking reservations with a minimum searching time, less cost, and maximum resource utilization and revenue for parking leaders. The offered system is based on a mixed-integer linear programming technique, with the objective of decreasing the cost of all drivers in the system and utilizing the parking resources with maximum abilities.

Pham, Tsai et al. in [20] proposed a parking algorithm to improve the efficiency of the smart parking system’s cloud and build an efficient architecture using the available internet-of-things technologies. The proposed system directly assists the drivers in finding empty parking spots with minimum cost by using new metrics to evaluate the driver parking cost in terms of the distance and total number of empty locations in each car park. This cost value was used to offer a solution to finding empty spaces, or suggesting a car park when the parking spaces are full. Hence, the probability of successful parking was improved, and the driver waiting time reduced.

In [21], a smartphone parking reservation system was proposed in a city center region to manage the reservation of curbside parking spots situated in different locations. It was built to reduce the social parking cost, which is equal to the weighted sum value of the cruising time for a driver to move from the current location to the parking spot location, and the walking times from the parking spot to the last destination. Hence, a simple reservation approach was presented, given the assumption of perfect data for both walking and cruising times, to reach the optimum allocation of the parking spots. Chen, Yin et al. also applied the Vickrey–Clark–Groves mechanism to identify the allocated spot and its fee in order to reduce the social cost and ensure that the driver will inform his last destination correctly.

#### 2.1.2. Parking Guidance and Information Systems Group

Geng and Cassandras, in [18], proposed a smart parking system for urban regions, which selects and reserves the optimal parking spot based on a cost function that combines both the parking cost and proximity to destination factors. They addressed the parking problem as a mixed-integer linear programming problem at each decision iteration in a time sequence. Therefore, they concluded that optimal allocation is obtained based on the current data and updated, with a guarantee that there are no reservation conflicts and no spots are assigned when the cost is greater than the nominal cost value. Therefore, the average time to locate the vehicle and the parking cost are reduced while ensuring the system is efficient for the whole process.

In [22], Tandon and Gupta proposed an online reservation tool for parking area, which aims to decrease the waiting time and parking cost for each car. Additionally, they developed an approach to evaluate the overall cost of the cars. This online reservation system showed that the overall cost is lower compared to other parking strategies. Pazos, Müller et al. in [23] proposed a mobile guidance software called SmartPark that helps drivers find the “optimal” parking spot. This depends on the available data of the parking systems, as well as other sensors. Hence, a video-processing smart camera and fixed magnetic on-road sensor were integrated with the SmartPark. The output data are available on a cloud based on the internet-of-things technology and updated automatically over a period of time.

Klappenecker, Lee et al., in [13], modelled a given parking lot as a continuous-time Markov chain. The parking lots utilize the advantage of vehicular network for obtaining a number of empty spots, arrival and parking rates, and capacity. However, the car navigation system is used to compute the probability of finding empty spots from the collected data, based on the arrival rate. Kokolaki, Karaliopoulos et al., in [24], formulated an information-assisted parking search problem as a selection game, with three variants (Bayesian, strategic, and preBayesian), given the normative prescriptions driver decisions. The bounds of the game are derived based on what the drivers can achieve by the rational strategy, which reduces the overall cost of their decisions. This game achieved better efficiency in terms of parking search process.

Li, Pei et al., in [25], used the concept of multiple-objectives decision-making theory to propose a smart parking guidance approach while ensuring three main decision parameters (i.e., parking fee, walk time, and the number of empty parking spots) and the driver specification. This considered the expected empty spots as a dominant objective to show the degree of complexity of finding empty spots. Then, they proposed a queueing theory-based theoretical approach to find the expected number of empty spots with different arrival rates, capacities, and service rate values. The advantages and disadvantages of the two groups based on this research are summarized in Table 1.

### 2.2. Algorithms for Smart Parking

In [26], the authors’s main objective was to find a solution to the path-planning problem in the street network, and then find the equilibrium point of this optimization problem. The streets in this problem have uncertain objectives. The authors used Dijkstra’s algorithm and Dempster–Shafer theory in the implantation of the parking problem. This was used to assist in the modeling of the uncertainty effect and finding the optimal path to the parking spot. The travel time variable was then evaluated based on the uncertain influencing factor for each street. Next, an approach was used to specify the best path based on the travel time values and using appropriate decision policies with respect to the user’s attitude.

Lejdel, in [27], proposed a smart parking approach based on the multi-agent system and genetic algorithm. It assists agents in fining the optimal parking spot, where an agent sends a parking request based on his location on the street and the parking and waiting time. However, the approach depends on four parameters: cost of parking, availability of a parking spot, traffic congestion, and the distance between the current location of the agent and the destination parking location. Hence, the proposed approach helps to achieve a better utilization of spot resources in a city as parking, and minimize the parking and waiting time. In [28], Li Y. et al. presented a game theoretic algorithm to investigate the search problem for finding empty spots in a parking lot and selecting the optimal one. This algorithm uses restricted data given by the parking lot, such as the number of vehicles and the layout of parking lot. Hence, the algorithm can work without any updates for the available parking facilities. However, authors in a large parking lot integrated the proposed algorithm with a sampling strategy to avoid the associated computational problem in the system.

In [29], the authors formulated and analyzed the games that arise in the use of strategic rational decision-making. Then, they investigated the parking search performance under such heuristics and compared the results. They also mathematically derived the conditions that result in better efficiency for the heuristic outputs in the single-objective problem. The results showed that, in such realistic scenarios, the deployment of heuristics results in a better parking search than when the agents use a strategically similar option with the game prescriptions. In [30], Li P. et al. presented a selection game for the parking lot search problem. To achieve the collaboration between vehicles, authors updated the strategy cost by using a new pricing policy. Each driver’s decision in the competing parking lot should pay more cost of opponent’s lost with respect to the rule to construct the Nash equilibrium with better efficiency for all competitors.

Mejri, A. et al. in [31] discussed the parking problem from two perspectives. First, they considered how the parking coordinators can utilize the distribution of spots and how to collaborate between them. Second, they modelled the parking problem as a congestion game, where the players of the game represent the vehicles which will choose the optimal parking garage, while ensuring minimum cost for the whole network. In [32], Mitsopoulou and Kalogeraki presented a crowdsourcing system that works to find the optimal paid parking spots for drivers based on their needs. Then, they developed an algorithm called ParkMatch, which searches for suitable solutions with respect to the driver’s requirements while continuing to update the parking state. Via a detailed simulation, the proposed system worked well under different parking providers’ price-policies and driver requirements.

Kokolaki and Stavrakakis, in [33], modelled the agents’ decision-making in terms of the parking spot search process, which was considered as the major cause of congestion problems. They studied the parking search problem as an instance of sequential search, and then presented a game approach based on the heuristic parking search strategies to identify an available parking spot with minimal driving and walking overhead. Mamandi, Yousefi et al., in [34], investigated two parking models in order to analyze the performance of the parking guidance system. The two models work based on the game theory concept and priority heuristic. In the game model, drivers are considered as the players and rational entity, and the search aims to increase their utilities. In the priority heuristic, the drivers’ features are considered to select the car park. The models achieved better efficiency compared with previous models in term of the number of on-road car park spots, difference in costs between on-road and private car parks, and the total number of drivers.

In [35], Du and Gong proposed a distributed and coordinated online parking mechanism (DCPM). It aims to minimize the parking congestion using a set of parking facilities in the central distinct by using the parking rules of the parking coordination set. To construct this mechanism, a stochastic Poisson game was introduced to implement competition among vehicles in the route, based on a set of parking facilities. The equilibrium point for this game was formulated using the drivers’ parking behaviour, represented by a multinomial logit model. Additionally, they developed a distributed algorithm to find the equilibrium point of this mechanism.

Krapivsky and Redner in [36] discussed the parking problem in a one-dimensional lot, where each vehicle enters the park at specific arrival rate and tries to park near the destination of origin, and the parked vehicle departs at rate equal to 1. However, the incoming driver cannot see if there are more suitable open spots outside the parked vehicles. They analyzed a set of strategies where the driver neglects the open parking spots within a specific distance. When the drivers employed this strategy, the resulted probability of parking at the optimal spot was maximal, with a risk threshold equal to 0.5, and the probability of parking at this spot is 0.25.

A Multi-Destination Vehicle Route Planning (MDVRP) approach was proposed in [37] to utilize the travel time for drivers. This approach consists of two items: a server in the cloud that utilizes the paths by identifying the best path to reach the destinations, and a mobile application for the drivers to present the real-time path and navigate the drivers to their destinations. MDVRP used a Time-Dependent Traveling Salesman algorithm, which looks for the fastest path to the next destination and then assigns the empty curbside parking spots in order to reduce the travel time for all drivers. In [38], Klandev, Tolevska et al. proposed a machine-learning regression approach for predicting the parking availability ratios. They used the garage occupancy and traffic congestion data collected from the public services as inputs to the approach, and then predicted the availability of parking spots in the same garage after sixty minutes. They tested the problem using sn XGBoost regression model and it achieved low error values, hence confirming the efficiency of the proposed approach.

Xiao, Lou et al., in [39], proposed an approach-based practical framework to expect the future occupancy from only the available historical information. The proposed framework consists of two stages: model parameters’ estimation stage, and occupancy prediction stage. In the predictive stage, they used a queuing approach to represent the occupancy variation of the parking facility. Indeed, they demonstrated the proposed framework using a continuous-time Markov queue (M/M/C/C); they also investigated other queuing models to check the validity of this framework. Both occupancy prediction and parameter estimation were constructed based on this queuing model. They also handled the change in day-to-day arrival and departure samples when applying the prediction and estimation models in the real world.

Previous research solved the parking problem as an optimization problem [24,26,27,33], game theoretic problem [28,30,31,34,35], queueing problem [39], and machine learning problem [38]. Different preferences were considered in this research, such as driving distance to the parking area, parking cost, physical positions, availability of parking resources, traffic congestion on the streets to the parking area, and driving time to the parking area. In our research, we constructed the parking equation based on five factors: traffic congestion, trip distance, availability of the parking spots, the waiting time on the lot gate, and the parking fees. The suitable path and parking spot will be selected based on the value of the parking equation. This value should utilize the available resources for distributing the loads on the parking lots, reduce the congestion traffic rates on all paths to the parking area, and minimize the driving time to the parking area.

## 3. Parking and Routing Approach

The routing and parking problem for finding the optimal parking spot is considered a multi-objective decision-making problem, as there are certain objectives that need to be satisfied, as well as some weights associated with those objectives based on different parking facilities and drivers’ preferences. Parking distances, walking distances, parking cost, physical positions, and the availability of parking resources are the main factors used for finding the optimal parking spot in the destination area, especially when the number of parking spots is limited and the traffic congestion is high. However, the availability of such parking resources is stated, as the probabilities of empty parking spots, which represent the ratio between reserved or the empty spots to the total capacity of the parking lot, or the ratio of the arrival cars to empty spots in this parking area. This section presents the problem, assumptions, and the proposed solution in terms of the above objectives.

### 3.1. Methodology

The parking and routing problem was previously addressed using different types of solution, such as Queueing models [12,13,40], time series models [11], neural networks and machine learning [9,10], and multivariate autoregressive model [14]. The multivariate autoregressive model is considered as one of the important models that can used to predict the availability of a parking resource in a given parking lots. Therefore, it can be used to solve the parking and routing problem by considering five main factors related to the parking facility, such as parking fee, accumulated path congestion, waiting time at the parking gates, driving distance, parking availability.

The availability of the parking spots in any parking lot can be modelled as a queueing system with capacity (c) and the dynamical variation in the empty spots (k) can be modelled using a Markov chain. Therefore, the transition probability of estimating empty spots can be defined in a closed form. Additionally, the expected empty parking spots are then evaluated based on the product total for the number of empty spots and their transition probabilities. Based on the previous description, how to first find the best path to the gate of the parking lot and choose the optimal parking spot in the parking lot based on a such decision-criteria, depends on both traffic and parking factors.

#### 3.1.1. Specifications

**Figure 1:**
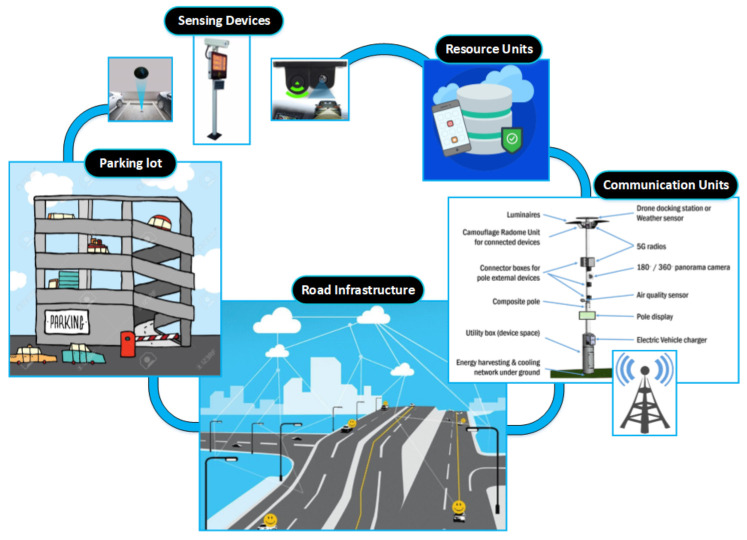
Basic components of the routing and parking system.

The parking lots are supported by different communication systems and sensing devices to sense and distribute the state of the parking spots. These sensing devices, such as ultrasonic sensors, image and video signal processors, radio frequency identification readers, and microwave radars, can be employed to monitor/sense the state of the parking spots based on a specific range. Moreover, they are connected to a server, which is used to store the collected data and can be remotely accessed using wireless or wired communication links. The factors of each parking lot, such as parking fee, GPS position, capacity, departure and arrival rate of the cars, and the number of reserved spots, are then sent to the parking resource central unit. Drivers can access this unit online and obtain the required information of the parking lot within the destination region. Additionally, the data stored in the central unit can be utilized to produce a real-time navigation for the drivers in the moving cars. This navigation process can be done using either portable navigation devices in the mobile or the car, or even using a central navigation unit. Figure 1 shows common components of any smart routing and parking system.

#### 3.1.2. Assumptions

The following is a list of assumptions have been made for studying the routing and parking problem:

- Historical data for parking lots are available;
- Historical data of the traffic congestion on the roads are available;
- GPS position of cars is available;
- Cars, parking-lots, and smart links communicate based on the available wireless network;
- Parking lots are supported by communication and sensing devices;
- Algorithm works based on a given parking area; any parking lot considers a candidate solution;
- The starting point of the car and the entry time to the parking area are defined by the driver.

### 3.2. Parking and Routing Algorithm

**Figure 2:**
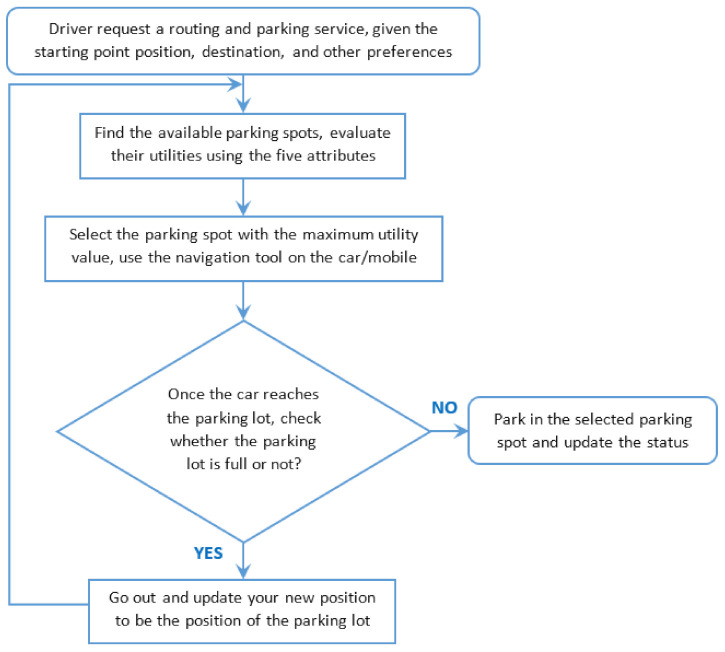
Diagram of the multiple objectives algorithm.

The selection of suitable parking spots and the best path for the drivers depends on a decision criterion with multiple objectives. The driver will choose his parking lot in the destination area and this will direct him from the source position to the selected parking spot based on the optimal values. Given that the positions of the cars and the available parking spots, and the use of the shortest path algorithm to find the best route to the parking spot. Hence, this parking problem relates to how to choose the parking resources required to achieve a common technique among a limited number of spots, with different factors such as parking fee, accumulated path congestion, waiting time at the parking gates, parking availability, and driving distance to parking. Figure 2 shows the flowchart of a simple multiple-objectives decision-based routing and parking algorithm.

For each driver, suppose that the current position is $p_{c}$, the destination position is $p_{d}$, and there are *n* parking spots in the parking lot, which are individually defined by $\left[P_{1},P_{2},\cdots ,P_{n}\right]$ for each driver in the parking model. The routing and parking problem can be modelled as a directed or undirected graph, where the parking lots are considered as the nodes of this graph, and the streets that link between these nodes represent the edges. The streets in the system model can either be one-way streets (i.e., directed) or two-way streets (i.e., undirected). If we have a set of parking lots (N) and each parking lot is defined by $P_{i}:i\to \left(1\;\leq \;i\;\leq \;n\right)$, suppose that its specifications can be determined by a set of variables, such as its parking fee, GPS coordination, capacity, arrival rate $\;\lambda_{i}$, number of reserved spots, and service rate $\;\mu_{i}$. We also have a set of decisions for selecting the suitable parking spot, which are defined by $d_{ij}$, where j is the decision criteria $j\to \left(1\;\leq \;j\;\leq \;m\right)\;$ values for $P_{i}$. The decision matrix ${(D}_{matrix})\;$ for all available decisions and its normalized matrix $\left({\bar{D}}_{matrix}\right)$ can be defined as follows, with respect to the parking spots

(1) $$\begin{array}{c} \;D_{matrix}=\begin{array}{c} P_{1}\\ P_{2}\\ \begin{array}{c} \vdots \\ P_{n} \end{array} \end{array}\left(\begin{array}{ccc} \begin{array}{c} d_{11}\\ d_{21} \end{array}\; & \cdots & \begin{array}{c} d_{1m} \end{array}\\ \begin{array}{c} \vdots \end{array}\; & \ddots & \vdots \\ d_{n1} & \cdots & d_{nm} \end{array}\right) \end{array}$$

(2) $$\begin{array}{c} {\bar{D}}_{matrix}\;=\begin{array}{c} P_{1}\\ P_{2}\\ \begin{array}{c} \vdots \\ P_{n} \end{array} \end{array}\left(\begin{array}{ccc} \begin{array}{c} {\bar{d}}_{11}\\ {\bar{d}}_{21} \end{array}\; & \cdots & \begin{array}{c} {\bar{d}}_{1m}\\ {\bar{d}}_{21} \end{array}\\ \begin{array}{c} \vdots \end{array}\; & \ddots & \vdots \\ {\bar{d}}_{n1} & \cdots & {\bar{d}}_{nm} \end{array}\right) \end{array}$$

where $d_{ij}$ and ${\bar{d}}_{ij}$ represent the jth objective of $P_{i}$ and the normalized of $d_{ij}$, respectively. However, the normalization process can be carried out based on two criterion [25] (i.e., the smaller the better or the larger the better) using the following equations

1. Minimization criterion: when you prefer to look for a minimum parking fee, this can be implemented by
  (3) $$\begin{array}{c} {\bar{d}}_{ij}=\;\frac{\left(D_{j}^{max}-d_{ij}\right)}{\left(D_{j}^{max}-d_{j}^{min}\right)} \end{array}$$
2. Maximization criterion: when you prefer to look for a maximum parking availability (# empty spots), this can be implemented by
  (4) $$\begin{array}{c} {\bar{d}}_{ij}=\frac{\left(d_{ij}-D_{j}^{min}\right)}{\left(D_{j}^{max}-D_{j}^{min}\right)} \end{array}$$

$D_{j}^{min}$ and $D_{j}^{max}$ represent the minimum and the maximum values of the decision criteria $D_{j}$, respectively. In this model (m=5), five key factors are considered as follows:

1. Accumulated path congestion;
2. Waiting time at the parking gates;
3. Driving distance to the parking area;
4. Parking availability;
5. Parking fee.

Since we have five factors that can be used to obtain the best route and the best parking spot, the overall objective function for the selected parking spot can be determined using the weighted sum of the five factors. Each driver has different weights $\left(w_{j}\right)$ for these factors and they represent the importance of the objective. The associated weights and the transition matrix are known based on our assumption and previous equations; the utilization of any parking spot $P_{i}\left(i=1,2,\cdots ,n\right)$ can be calculated at each destination node or junction point on the street using the following equation, where the final destination node represents the parking spot:

(5) $$\begin{array}{c} U_{i}=\sum_{j=1}^{m}w_{j}\;{\bar{d}}_{ij} \end{array}$$

**Figure 3:**
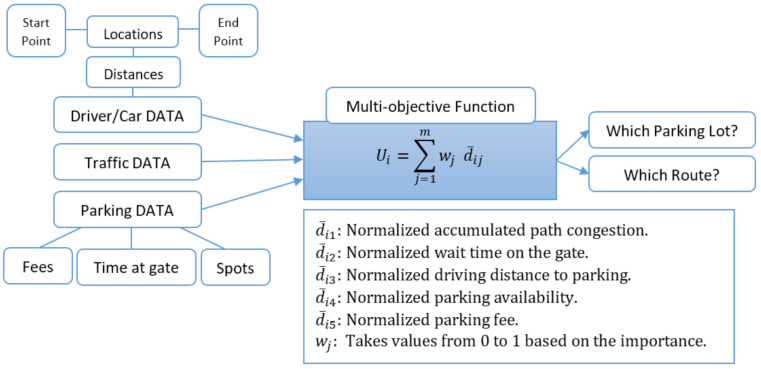
Description of the multi-objective function.

Figure 3 shows the inputs of the algorithm and its target outputs with descriptions of the decision coefficients and weight.

After using the above weighted equation, the parking spot with a higher-objective function will be selected as the final destination, since this will achieve a better performance for the whole parking system. Then, the navigation device, either on the car or on the mobile, will guide the driver to this parking spot. It is easy to estimate $d_{i2}$, $d_{i3}$, and $d_{i5}$ for each parking spot $P_{i}$ by using the physical distance to the destination, parking fee rate, speed, and the parking time. On the other hand, estimation of both the parking availability and the minimum congestion rate are still challenging tasks and need more investigations. Hence, the main steps to find the best parking spot are as follows:

1. The starting point and the arrival time to the destination point are given;
2. Predicting the availability of the parking spots in the parking lots based on historical data;
3. Predicting the minimum congestion path value to the parking lot based on historical data;
4. Evaluating the objective function for each parking spot after point 2 and point 3;
5. Selecting the parking spot with highest objective function.

Based on the above description, point 2 and point 3 require information about the estimated arrival time to the parking area for predicting the future empty spots, as well to the junctions in the way to the parking area for estimating the future congestion values.

#### 3.2.1. Queueing Model for the Availability Objective

**Figure 4:**
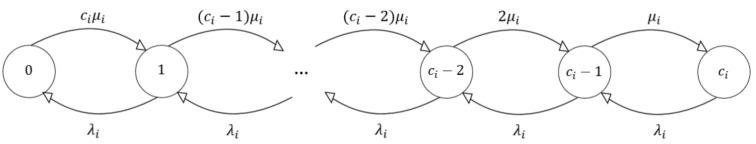
Markov model for parking lot $P_{i}$ with capacity $c_{i}$.

A queueing model is required to estimate the availability of the parking spots in the parking lot; we can model this factor based on the arrival and service rate of the cars and the capacity of the parking lot. The arrival and departure of the cars in the parking lot $\left(P_{i}\right)$ follows the Poisson distribution with arrival rate $\lambda_{i}$, service rate $\mu_{i}$, and capacity $c_{i}$. Hence, we can also design the dynamical variation in the empty spots for every parking lot as (M/M/c/c) queueing problem [41], provided that, if there is no empty spot, the driver is not permitted to wait inside the parking lot to ease the departure of the parked cars. The features of the M/M/c/c queueing model are described as follows:

1. Arrival rate $\lambda_{i}$ and service rate $\mu_{i}$;
2. State of the empty spots x(t)(=0,1,2,...,c) in the parking lot at time t(>0);
3. Driving duration from the starting point to the parking spot is τ(>0);
4. Estimating the empty spots at (t+τ) in the parking lot can be represented by Markov process with continuous time discrete state, as in Figure 4, where the number of empty spots at time *t* or the state of the queue is $s_{i}$, and the transition probability of finding $s_{j}$ empty spots is $p_{s_{i}s_{j}}\left(t,\;t\;+\;\tau \right)$. By assuming that the process is homogeneous, this leads to an independent transition probability to the start time *t*, and its value is $p_{s_{i}s_{j}}\left(t,\;t\;+\;\tau \right)=p_{s_{i}s_{j}}\left(\tau \right)$. Based on the (M/M/c/c) queueing model described in [13,41], the probability can be expressed by $p_{s_{i}s_{j}}\left(\tau \right)=e^{Q\tau }$, where *Q* represents the transition probability matrix and its value is
  $$\begin{array}{c} Q=\left[\begin{array}{c} -c\mu \\ \lambda_{i} \end{array}\;\;\begin{array}{c} c\mu \\ -\left(\lambda_{i}+\left(c-1\right)\mu_{i}\right)\\ \begin{array}{c} \lambda_{i} \end{array} \end{array}\;\;\begin{array}{c} \left(c-1\right)\mu_{i}\\ \begin{array}{c} -\left(\lambda_{i}+\left(c-2\right)\mu_{i}\right)\\ \vdots \\ \begin{array}{c} \lambda_{i} \end{array} \end{array} \end{array}\;\;\begin{array}{c} \begin{array}{c} \left(c-2\right)\mu_{i}\\ \begin{array}{c} -\left(\lambda_{i}+\mu_{i}\right)\\ \lambda_{i} \end{array} \end{array} \end{array}\;\;\begin{array}{c} \begin{array}{c} \begin{array}{c} \mu_{i}\\ -\lambda_{i} \end{array} \end{array} \end{array}\right] \end{array}$$

#### 3.2.2. Pseudo Code

Based on the description of the objective function and the selected variables, we can summarize the proposed algorithm using a pseudo code as illustrated in Algorithm 1. It consists of two main stages, as follows:

1. **Stage one:** contains the simulation setting of the algorithm, such as initialization of the variables, congestion rates for the main roads, loads at the gates of the parking lots, distances between the junctions and the parking gates, arrival and service rates, and the availability of the parking spots;
2. **Stage two:** contains the main function of finding the best route to the parking spot, with minimum time, less load on the streets and parking gates as described in Equation (5). The simulation scenario studies the five main prayer time periods, Fajr (dawn), Dhuhur (noon), Asr (midday), Maghreb (sunset), and Isha (night), with different availability and congestion rates. These prayer times are denoted in the remainder of the manuscript as PR1, PR2, PR3, PR4, and PR5, respectively.

**Algorithm 1** Pseudo code of the Parking and routing algorithm. 1:**procedure**Initialization 2:  Define the input variables 3:  Define the outputs: availability, congestion, drivingTime, bestPath and bestSpot 4:  Set the weights based on the case(= 1,2,3) 5:  Set the distances between all junctions and between the junctions and the parking lots 6:  Initialize arrival rate, departure rate, traffic intensity value 7:  Initialize congestion rates for all links, waiting time on the gates 8:  Initialize availability rate for all parking lots based on the capacity 9:  Set time slots to 5 (PR1, PR2, PR3, PR4 and PR5)10:  Set the simulation time for each time slot11:  Divide the simulation time into *T*’s12:  Define the parking lots (P1, P2, P3, P4, P5 and P6)13:  Define the cost of the parking lots14:  Define the states of the transition matrix based on the available spots15:**end procedure**16:**procedure**TransitionMatrix(parkingLot,arrivals,departures)17:  updates the empty spots in the parking lot18:  update the ratio of the states in the matrix19:  Return updated transition matrix20:**end procedure**21:**procedure**ParkingRoutingAlgorithm22:  Call Initialization procedure23:  Specify the time slot or study the five slots24:  **for** each PR in timeSlots **do**25:    **for** each *T* in simulationTime **do**26:      Generate arrivals based on the arrival rate and traffic intensity27:      **for** each arrival in arrivals **do**28:        Specify the starting point29:        **for** each *P* in parkingLots **do**30:          Get all path from starting point to the parking lot31:          Call TransitionMatrix procedure for finding the availability rates32:          **for** each Path in Paths **do**33:            Get the congestion rate, waiting time at the gates34:            Get waiting time at the gate35:            Get the distance to the parking lot and the cost36:          **end for**37:          Generate departures based on the departure rate of the parking lot38:        **end for**39:        Find the objective function for all paths to the parking lots40:        Define the best path and the best spot for this arrival based on the objective function41:        Update all input variables based on the best values42:        Send the best values to the arrival and store them in the outputs43:      **end for**44:    **end for**45:  **end for**46:**end procedure**

## 4. Simulation Setup and Performance Metrics

Smart parking is an important application as it saves a significant amount of parking spot search time, minimizes on-street traffic, and reduces pollution in busy downtown areas. We evaluate the performance of the proposed routing and parking algorithms by means of simulation study. We also studied the problem based on the suggested queueing model for the availability factor and the Markov chain for the dynamic changes in the available parking spots. In this section, we introduce the candidate area for the simulation. Then, we describe the simulation setting used to build and evaluate our algorithm. After that, we mention the performance evaluation metrics and show the results based on the simulation setting for five time periods (PR1, PR2, PR3, PR4 and PR5).

### 4.1. Case Study

We consider the application of the proposed algorithms in one of the busiest cities in the world during specific religious seasons. The city of Al-Madinah in Saudi Arabia is the second holiest Muslim city in the world, in which lies the tomb of prophet Mohammad (peace be upon him) and his holy mosque (the Haram). In some seasons, an estimated number of over one million pilgrims per night perform their prayers. Many of the pilgrims commute using their own personal vehicles, which creates significant demand for the infrastructure and organizing authorities. A smart parking system for the Haram area is needed to overcome many of the issues/problems that arise at various times of year due to the large number of visitors.

**Figure 5:**
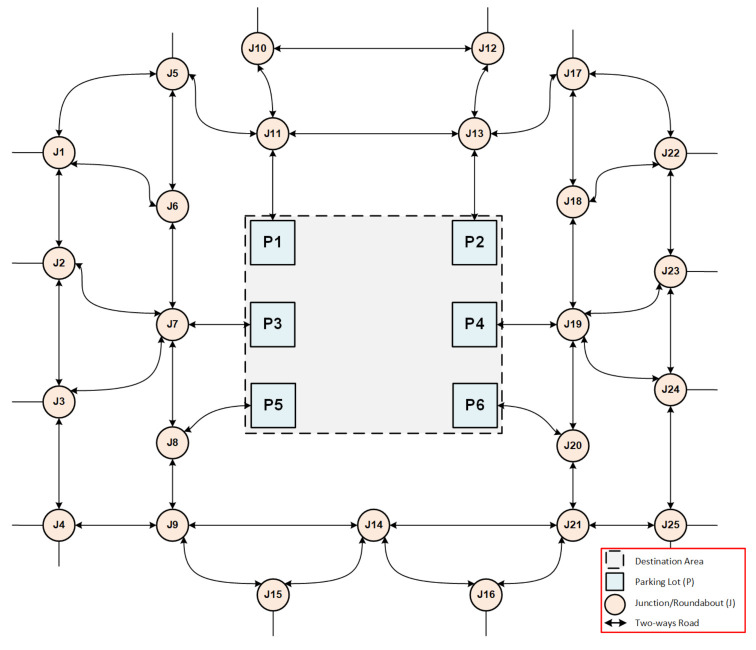
Simulation area.

There have been several attempts to provide parking solutions to visitors, such as showing signs at the entrance of the Haram parking zone informing incoming visitors of the availability/unavailability of the underground parking spots. The parking space has six entrances in various directions (East, West, North, South). Figure 5 shows an undirected graph, which represents the underground parking lots of the Haram. Each link between two junctions or between junction and parking lot represents a distance of 1.5 km.

### 4.2. Simulation Setting

The candidate area is located in the middle of Al-Madinah city and can be reached through a set of two-way streets. Each street has a different congestion rate value and the route consists of set of junctions before reaching the parking area. The parking area has an area of 200,000 square meters on two underground levels with a total of 11 parking lots. Each lot has a separate electronic gate at its entrance and exit. To help follow the simulation of different parking scenarios for a set of periods, Table 2 summarizes the required parameters that can be employed and changed for building the simulation scenarios, including the factors of each parking lot, the congestion rate of the streets, the arrival and departure rate of cars in each parking lot, and others. Assume there are, at most, six parking lots in the destination area, and each parking lot has a separate gate for entrance/exit, which is different from other parking lots.

### 4.3. Performance Metrics

We study the performance of the proposed smart routing and parking algorithm at the central region of the prophet Mohammad holy mosque (Al Madinah Haram) with different simulation scenarios. The simulation consists of five main slots based on the local prayer times (PR1, PR2, PR3, PR4 and PR5) and the simulation time of each slot is 45 min. As an approximation, we consider a discrete time domain in our simulation scenarios. Vehicles visiting the central region are assumed to use the smart routing and parking system.

In this work, the Python programming language is used for implementing the routing and parking system and performing numerical calculation to evaluate the objective function values based on five factors (i.e., the parking fee, the cumulative path congestion, waiting time at the parking gates, parking availability, and the driving distance to the parking area). Each factor has a specific weight, affecting the overall objective function. The user preference is demonstrated by setting these weighting values. The simulation is done for both low and high traffic rates. Three cases, according to user preference, have been considered, as illustrated in Table 3. Each case is represented by different weighting values for the five factors.

The investigation of these factors with different weights shows the benefit of using such a smart routing and parking algorithm. This saves the search time for finding the best spot, reduces the congestion on the roads and the parking gates, and adjusts the loads on the parking lots. We use the following performance metrics to evaluate the performance of the proposed algorithm with various preferences for low and high traffic rates and at different time slots.

#### 4.3.1. Average Availability Ratio of the Parking Lots

This metric is defined as the number of empty spots at a specific simulation time t, divided by the total number of the spots in the parking lot. Since the destination area is studied during specific simulation periods, we need to calculate the average rate of the empty spots for all time slots in each simulation period. In this algorithm, if the parking lot is full, the user’s request is rejected, and they need to update their new locations and restart the search process. The availability ratio for the parking lot is investigated in our simulation in case 1, case 2, and case 3.

#### 4.3.2. Average Congestion Rate

The rate value represents how much the paths to the destination point are congested in the simulation period. To study this metric, we need to track the number of vehicles in each link by users, to reach their parking lots. So, it is important to reduce the traffic congestion resulting from the routing and parking algorithm. This can also be calculated by using the average of the standard deviation of the guided users to the parking lots during the simulation period. The congestion objective of the path is investigated in our simulation in case 1, case 2, and case 3.

#### 4.3.3. Average Driving Time

Driving time is defined as the required time for the user’s current location to the optimal assigned parking spot. The locations of both the start- and endpoints are given in the simulation, but other factors need to be set depending on the objective function. Therefore, all possible routes need to be investigated, from the starting point to the destination point, and the average driving time can then be calculated during a specific simulation period. This is defined by the sum of the driving times of all users divided by the number of users in the simulation. The driving time to the parking lot is investigated in our simulation in case 1 and case 2.

## 5. Results and Discussion

In this section, we discuss the simulation results of the routing and parking algorithm. The algorithm selects the best path and parking spot while distributing the load on the parking lots and its gates, and adjusts the congestion rates on all paths. The simulation scenarios are executed based on the setting in Table 1. We studied the simulation under three cases with low and high traffic intensity rates, as shown in Table 2. In this paper, the effectiveness of the algorithm was evaluated by using three metrics for different time periods (PR1, PR2, PR3, PR4 and PR5), as follows.

### 5.1. Average Availability Ratio

**Figure 6:**
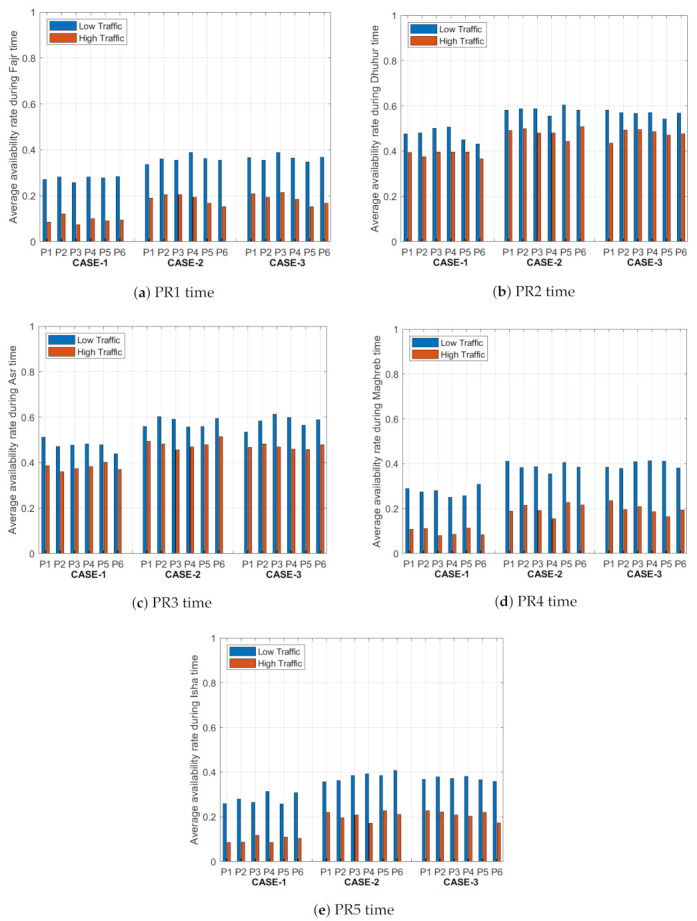
Average availability rates of the six parking lots (P1, P2, P3, P4, P5 and P6) for the five time periods (PR1–PR5).

The average availability ratio describes the average parking availability rate of the six parking lots in the system (P1, P2, P3, P4, P5, and P6) for the three cases under low and high traffic intensity. These parking lots consider the destination point of the driver, which can be reached through different paths. The algorithm tries to balance the distribution of the vehicles on the six parking lots based on the objective function. A compromise is reached between the congestion rate of the path, the waiting time at the gate of the parking lot, distance to the parking lot, availability spots in the parking lots, and the cost of the parking spot. Figure 6 shows the average availability rates of the parking lots for the five time periods. As mentioned before, the availability rate can be modelled using a queueing theory, where the states represent the number of empty spots in each parking lot (P1, P2, P3, P4, P5, and P6). The states are illustrated as follows: state 0 represents zero parking spot, state 1 represents from 1 up to 120 parking spots, state 2 represents from 120 up to 240 parking spots, state 3 represents from 240 up to 360 parking spots, state 4 represents from 360 up to 480 parking spots, and state 5 represents from 480 to 600 parking spots.

From Figure 6, The availability rate of the parking lots depends on the number of arrivals, where the arrival rate depends on the traffic intensity and the capacity of the parking lot. For low traffic intensities, the availability rate in the parking lot for any time period is high compared with the high traffic intensities. PR1, PR4 and PR5 periods in case 1, case 2 and case 3 have more traffic and arrivals compared with PR2 and PR3 periods due to the increase in the number of drivers visiting this area. The main difference between the three cases is that the weight values of the availability rate in the objective function change from 0.2 in case 1, to 1/3 in case 2, and 0.4 in case 3. As result, the availability rate for case 3 is higher than case 2 and case 1 due to the weight value, which makes this factor more dominant in improving the objective function and acheiving a better availability rate for the parking lot. In the PR1 period, under case 1, the standard deviation of the availability rates for the six parking lots is around 0.010 under low traffic and 0.016 under high traffic. However, for case 2, the standard deviation equals 0.017 under low traffic and 0.021 under high traffic. For case 3 in the same period, the standard deviation equals 0.015 under low traffic and 0.024 under high traffic. The same conclusion is reached for other time periods, where the maximum standard deviation equals 0.029 and the minimum standard deviation equals 0.009 under low traffic, and the maximum standard deviation equals 0.027 and the minimum standard deviation equals 0.010 under high traffic. In conclusion, when the traffic intensity value is low, the parking lot has enough empty parking spots for incoming drivers. On the other hand, when the traffic intensity is high, the probability of finding an empty spot will be low. We also notice from the low values of the standard deviation that the vehicles are distributed in a proper way to all parking lots.

### 5.2. Average Congestion Rate

**Figure 7:**
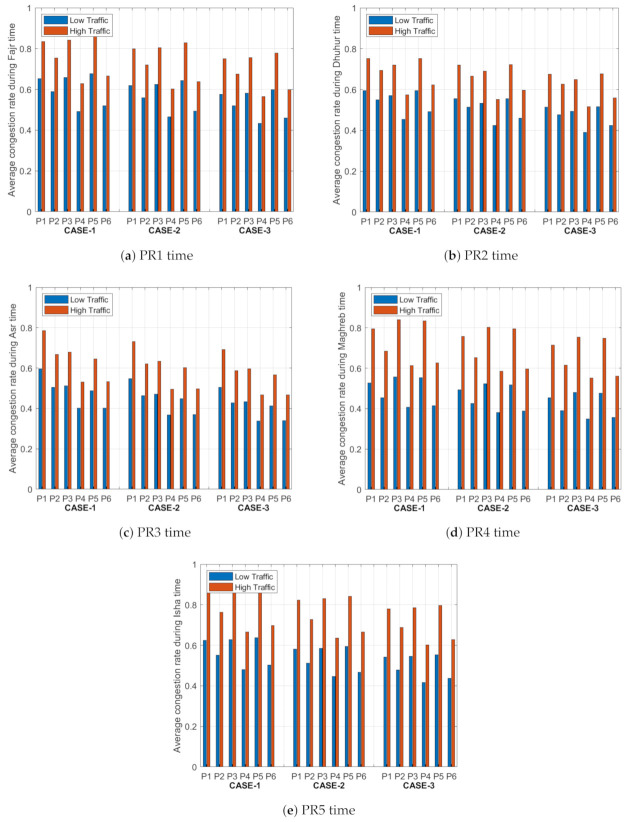
Average congestion rates for all paths to the six parking lots (P1, P2, P3, P4, P5 and P6) for the five time periods (PR1–PR5).

The average congestion rate describes the traffic congestion for all paths between the starting point and the six parking lots in the system (P1, P2, P3, P4, P5, and P6) for the three cases under low and high traffic intensity values. The driver can reach the parking lots using different paths. Our goal is to reduce the congestion rate on the paths by leading the vehicles to take different paths to the parking area based on the objective function. Figure 7 shows the average traffic congestion rates of the links between the start point and the parking lots for the five time periods and with different weight values.

From Figure 7, the traffic congestion rate is directly proportional to the number of driving vehicles in the path to the parking lot and the driving distance: the higher the traffic intensity, the higher the congestion rate. We change the weight value of the congestion rate in the objective function to investigate the behaviour of the congestion factor based on the three cases (i.e., case 1, case 2, and case 3). We notice that by increasing the weight of the congestion rate factor over other factors, it will dominate the values of the objective function and then distribute the vehicles on other paths based on the best objective function. We select the weight values of the congestion rate in the objective function to be 0.2 in case 1, to 1/3 in case 2, and 0.4 in case 3. Based on these values, the updated congestion rate for case 3 is less than case 2 and case 1 due to the weight value, which improves the objective function and then achieves a better distribution for the traffics on all paths to the parking area at any time period.

In PR1 period under case 1, the standard deviation in the average congestion rates for all paths to the parking lots is 0.078 under the low traffic and 0.099 under the high traffic. However, in the second case (i.e., case 2), the standard deviation equals 0.074 under low traffic and 0.095 under high traffic. For case 3, in the same period, the standard deviation equals 0.069 under low traffic and 0.090 under high traffic. The same conclusion was reached for other time periods: we found that the maximum standard deviation equals 0.078 and the minimum standard deviation equals 0.051 under low traffic, and the maximum standard deviation equals 0.103 and the minimum standard deviation equals 0.066 under high traffic. As a result, increasing the traffic on the paths will increase the probability of making paths more congested compared with low traffic values. From the standard deviation values of the traffic congestion rate, we can notice that the weight value will minimize the standard deviation value in case 3 compared with case 2 and case 1, and make it more stable, since the system will force the vehicles to select their paths to the parking area in a way that will distribute the traffic on all paths.

### 5.3. Average Driving Time

**Figure 8:**
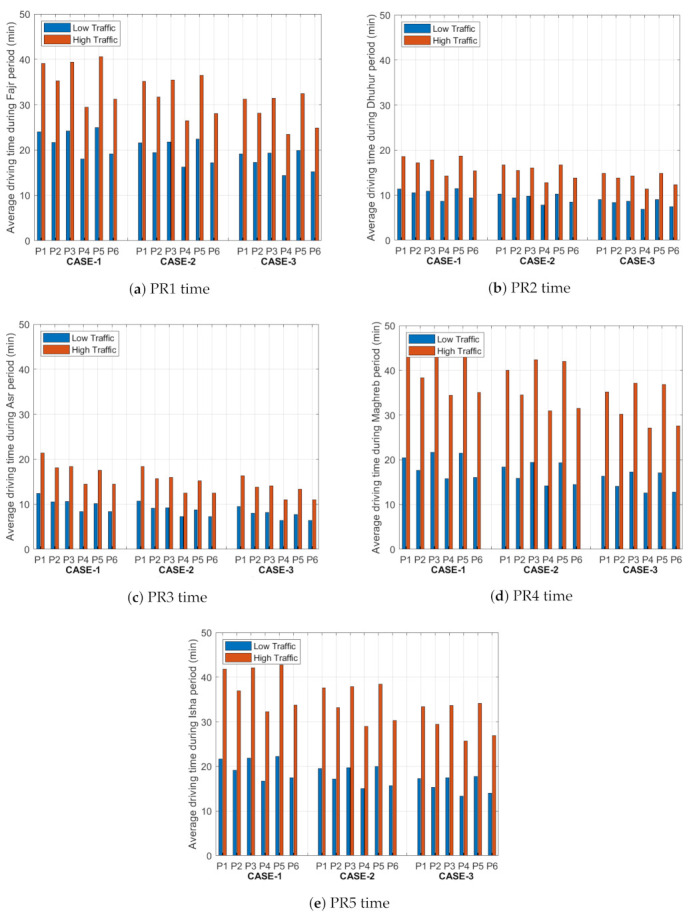
Average driving time (minutes) from the starting point to the six parking lots (P1, P2, P3, P4, P5 and P6) for the five time periods (PR1–PR5).

Average driving time represents the time it will take for the driver to travel from the starting point to the parking lot. This depends on the availability rate of the parking lots and the congestion traffic of the paths. This metric is studied under both low and high traffic values for the selected three cases. Usually, the driver selects the shortest path to the parking area; however, the system will not give him this option in all time periods unless this path is less congested and the objective function gives him a better value for the parking area compared with the remaining paths. We investigated the driving time of the trip for three cases during the five time periods under low and high traffic, and the outputs as shown in Figure 8. From Figure 8, we can see that the driving time to the parking area in case 1 during the PR1 time is higher than in PR2 and PR3 times, and it has similar values to PPR4 and PR5 times due to the similar rate values of traffic on the paths and the availability rates in the parking lot in these periods of time. A similar trend is achieved for other cases, but with lower driving times due the fewer less congestion rates and more spaces in the parking lots for the five time periods (PR1–PR5).

In the PR1 period under case 1, the standard deviation in the average driving time for all paths to the parking lots is 2.866 min under low traffic and 4.653 min under high traffic. However, in case 2, the standard deviation is 2.583 min under low traffic and 4.191 min under high traffic. In case 3, for the same period, the standard deviation equals 2.291 min under low traffic and 3.727 min under high traffic. From these values, we can notice that the increase in the weight values in the objective function causes a small reduction in the driving time from case 1 to case 2, and the same result from case 2 to case 3. The same conclusion is achieved for other time periods; we found that the maximum standard deviation equals 2.866 min and the minimum standard deviation equals 0.898 min under low traffic, and the maximum standard deviation equals 5.790 min and the minimum standard deviation equals 1.444 min under high traffic. To summarize, the travel time decreases with the increment in weight for both the congestion rate and the availability rate. Since this step means that the objective function is dominated by the two factors, it will generate the best path and parking spot for the driver in a way that will maximize the utilization of the resources.

Based on the simulation results and discussion, the algorithm provides load balancing on the paths and distributes the vehicles on the parking lots. Each of the three cases provides good results in terms of the weight values and which factor is the most weighted. In many cases, case 3 has better results in reducing the overall traffic congestion in all paths to the parking lots, increasing the empty spaces in parking lots, and reducing the driving time.

## 6. Comparison with Another Algorithm

We compared the proposed algorithm with another smart parking guidance algorithm, implemented in [25]. We began by describing the algorithm and its characteristics, and then showed the equations of both algorithms. Next, we compared [25] with our algorithm in terms of the availability ratios, congestion rates, and the required driving time to the destination point.

### 6.1. Description of the Selected Algorithm

This algorithm is based on multiple-attributes decision-making (MADM) theory. The decision factors in this algorithm are: the driving duration, the parking fees, and the availability of parking spots in the parking lot, along with a set of preferences. The availability of parking spots factor was considered as the dominant objective for showing the complexity degree of finding empty spots. A queueing model, used to find empty spots for different arrival rates, capacities and service rate values, was also proposed.

### 6.2. Characteristics of Both Algorithms

In our algorithm, the multi-objective function consisted of five factors (i.e., accumulated path congestion $\left(d_{i1}\right)$, waiting time at the parking gates $\left(d_{i2}\right)$, driving distance $\left(d_{i3}\right)$, parking availability $\left(d_{i4}\right)$, and parking fee $\left(d_{i5}\right)$), and each factor has a weight value $\left(w_{i}\right)$ between 0 and 1, as illustrated in Figure 3 and in the following equation

(6) $$U_{i}=w_{1}*d_{i1}+w_{2}*d_{i2}+w_{3}*d_{i3}+w_{4}*d_{i4}+w_{5}*d_{i5}$$

In [25], the function depends on three factors (i.e., driving duration $\left(do_{i1}\right)$, parking fees $\left(do_{i2}\right)$, and availability of parking spots $\left(do_{i3}\right)$), and each factor and each factor has a weight value $\left(wo_{i}\right)$ between 0 and 1, as follows

(7) $$Uo_{i}=wo_{1}*do_{i1}+wo_{2}*do_{i2}+wo_{3}*do_{i3}$$

To compare between the two algorithms, we set the weight values of our algorithm to be $\left(w_{1}=w_{2}=w_{3}=w_{4}=w_{5}=1/5\right)$, as in CASE-1, and set the selected algorithm as $\left(wo_{1}=wo_{2}=wo_{3}=1/3\right)$.

### 6.3. Results of Both Algorithms

We used three metrics (i.e., the availability ratios, congestion rates, and driving time) to compare between the two algorithms, with low and high traffic rates and for different time periods (PR1, PR2, PR3, PR4 and PR5), as follows

#### 6.3.1. Average Availability Ratio

**Figure 9:**
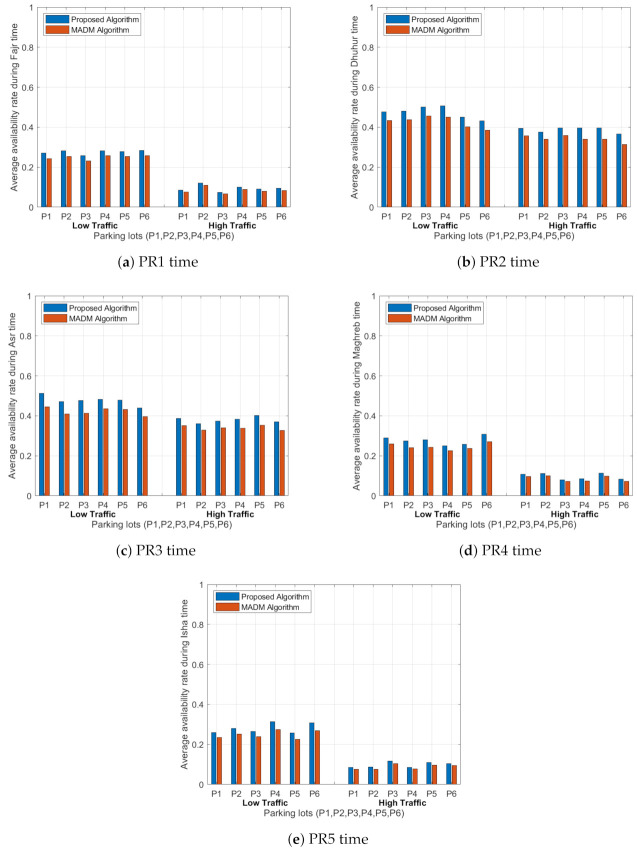
Average availability rates of the six parking lots (P1, P2, P3, P4, P5 and P6) for the five time periods (PR1–PR5) for both algorithms under low and high traffic rates.

Figure 9 shows the average availability rates of the parking lots (P1, P2, P3, P4, P5, and P6) for the five time periods for the proposed algorithm and the MADM algorithm under low and high traffic intensity values. In case of low traffic, the availability rates for all parking lots in the proposed algorithm are higher than the MADM algorithm, and these values are decreased in case of high traffic. PR1, PR4 and PR5 periods have more traffic and more arrivals compared with PR2 and PR3 periods, due to the increase in the number of drivers who visit this area for both algorithms. In the PR1 period, in the proposed algorithm, the standard deviation in the availability rates for the six parking lots is around 0.0101 under low traffic and 0.0161 under high traffic. However, in the MADM algorithm, the standard deviation equals 0.0103 under low traffic and 0.0144 under high traffic. The same conclusion was reached for other time periods, where the maximum standard deviation equals 0.0283 and the minimum standard deviation equals 0.0101 under low traffic, and the maximum standard deviation equals 0.0161 and the minimum standard deviation equals 0.0132 under high traffic in the proposed algorithm. In the MADM algorithm, the maximum standard deviation equals 0.0284 and the minimum standard deviation equals 0.0102 under low traffic, and the maximum standard deviation equals 0.0159 and the minimum standard deviation equals 0.0111 under high traffic. In conclusion, both algorithms try to equally distribute the vehicles in the parking lots, and the variation in the availability values between the two algorithms is very small for this network.

#### 6.3.2. Average Congestion Rate

**Figure 10:**
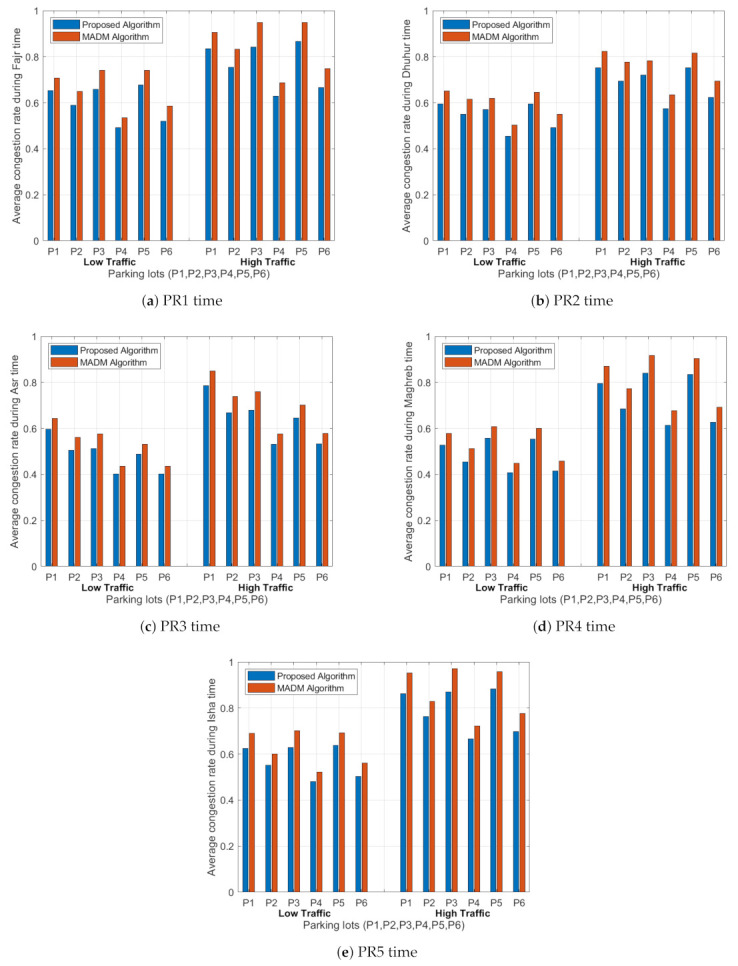
Average congestion rates for all paths to the six parking lots (P1, P2, P3, P4, P5 and P6) for the five time periods (PR1–PR5) for both algorithms under low and high traffic rates.

Figure 10 shows the average traffic congestion rates of the links between the start point and the parking lots (P1, P2, P3, P4, P5, and P6) for the five time periods for the proposed algorithm and the MADM algorithm under low and high traffic intensity values. It shows that the traffic factor depends on the number of driving vehicles in the path to the parking lot and the driving distance, where the higher traffic intensity has a higher congestion rate for both algorithms. Additionally, the average traffic congestion rates in the proposed algorithm are lower compared with the MADM algorithm results. In the PR1 period, in the case of the proposed algorithm, the standard deviation in the average congestion rates for all paths to the parking lots is 0.0778 under low traffic, and 0.0994 under high traffic. However, in the MADM algorithm, the standard deviation equals 0.0853 under low traffic and 0.1091 under high traffic. The same conclusion was reached for other time periods: we found that the maximum standard deviation equals 0.0778 and the minimum standard deviation equals 0.0578 under low traffic, and the maximum standard deviation equals 0.1034 and the minimum standard deviation equals 0.0727 under high traffic in the proposed algorithm. In the MADM algorithm, the maximum standard deviation equals 0.0853 and the minimum standard deviation equals 0.0590 under low traffic, and the maximum standard deviation equals 0.1091 and the minimum standard deviation equals 0.0745 under high traffic.

#### 6.3.3. Average Driving Time

**Figure 11:**
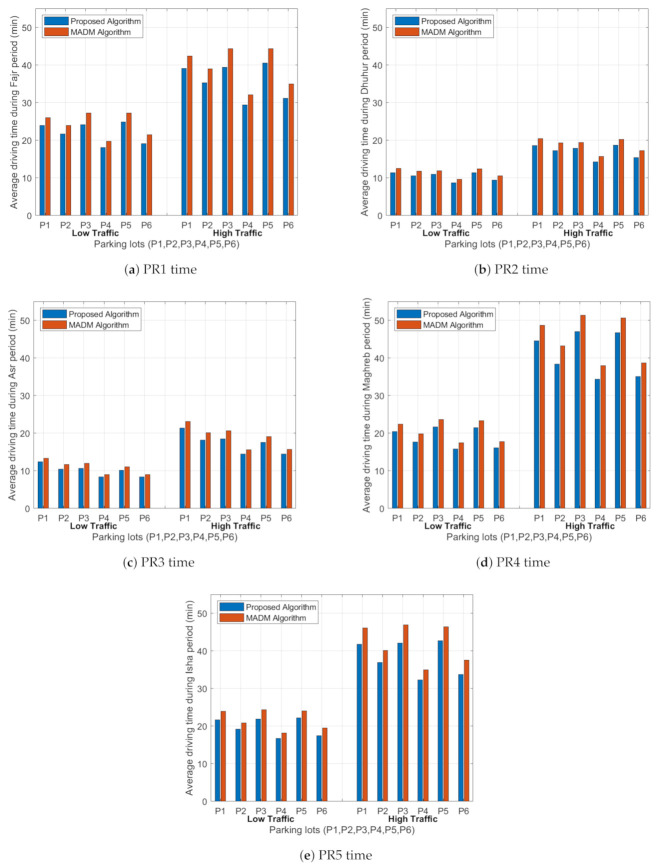
Average driving time (minutes) from the starting point to the six parking lots (P1, P2, P3, P4, P5 and P6) for the five time periods (PR1–PR5) for both algorithms under low and high traffic rates.

Figure 11 shows the driving time of the trip between the start point and the parking lots (P1, P2, P3, P4, P5, and P6) for the five time periods for the proposed algorithm and the MADM algorithm under low and high traffic intensity values. We can see that the driving time to the parking area in the proposed algorithm for PR1 is shorter compared to the values given in the MADM algorithm, and a similar trend is achieved for other time periods. With lower congestion rates, the drivers can reach the parking area in a shorter time, and more spaces will be available in the parking lots compared to the case of high congestion rates. In the PR1 period, using the proposed algorithm, the standard deviation in the average driving time for all paths to the parking lots is 2.866 min under low traffic and 4.653 min under high traffic. However, in the MADM algorithm, the standard deviation is 3.139 min under low traffic and 5.107 min under high traffic. The same conclusion was achieved for other time periods: we found that the maximum standard deviation equals 2.866 min and the minimum standard deviation equals 1.111 min under low traffic, and the maximum standard deviation equals 5.790 min and the minimum standard deviation equals 1.804 min under high traffic in the proposed algorithm. In the MADM algorithm, the maximum standard deviation equals 3.139 min and the minimum standard deviation equals 1.133 min under low traffic, and the maximum standard deviation equals 5.959 min and the minimum standard deviation equals 1.849 min under high traffic.

The proposed algorithm outperforms the MADM algorithm in the three metrics for the five periods. Additionally, the findings of the proposed algorithm show the feasibility of using this algorithm for managing traffic on the roads and equally distributing vehicles among the available parking lots.

## 7. Conclusions and Future Work

In this paper, we presented a smart routing and parking algorithm that balances the load on available parking lots, reduces the congestion traffic rates, and minimizes the driving time to the destined parking spot. The presented algorithm is based on a multi-objective optimization strategy to satisfy the transportation authorities and drivers’ objectives. The multi-objective optimization function includes five weighted factors (i.e., traffic congestion rate, trip distance, availability rate in the parking lots, parking gate waiting time, and the parking cost). The algorithm produces a parking spot assignment that ensures an overall balanced congestion rate on route to various parking lots, as well as a balanced parking lot utilization. We tested the algorithm on a very large parking facility in Al Madinah city in Saudi Arabia. The effect of the five factors of the multi-objective function is evaluated in three cases, based on different weight settings and under low and high traffic intensity rates. The simulations showed the effectiveness of the proposed algorithm for balancing the parking lot utilization, reducing the congestion traffic rates on all routes to the parking spot, and minimizing the driving time to the parking spot. Additionally, the proposed algorithm outperforms the MADM algorithm in the three metrics for the five periods. As a future work, on-street parking could also be included in our simulation tests for better balance and more evenly distributed parking with less traffic congestion.

## Figures and Tables

**Table 1:** Pros and cons of the reservation-based group and guidance-based group.

Parking System Group	Pros	Cons
Parking reservation systems	Pre-reservation is required. Availability of empty parking spots is guaranteed. Implementation is soft and straightforward. Easy and faster payment process through the system. Online reservation parking system is always available. You can get insights about the parking area in advance.	It provides static paths to the parking areas. Reservation system means no free parking. It needs to be online to do the reservation process. It might face accessing problems for the reservation system if there are too many customers.
Parking guidance and information systems	Cost-effective and efficient. Real-time data availability. Optimal utilization of parking spots. It can be implemented in a distributed manner, and it can be constructed for public and private parking spots. It saves time, easy to use, and it increases the comfortability of the driver.	It causes some competitions between the drivers on the parking spots. It is more complicated than the reservation system in the implementation part.

**Table 2:** Setting of the simulation parameters.

Parameter	Value
Number of parking lots $\left(P_{i}\right)$	6
Total spots per parking lot $\left(c_{i}\right)$	600
Initial availability rate	0.8
Time slot	5: PR1, PR2, PR3, PR4 and PR5
Simulation period per slot	45 min for each slot
Fees (SAR/hour) for the spot	5 for spot number [1–120), 4.5 for [120–240), 4 for [240–360), 3.5 for [360–480), and 3 for [480–600]
Departure rate $\left(\mu_{i}\right)$	0.2
Traffic intensity $\left(\sigma_{i}\right)$	Low: uniform (0.4,1) High: uniform(1,1.6)
Arrival rate $\left(\lambda_{i}\right)$	$\lambda_{i}=\sigma_{i}\times \mu_{i}\times c_{i}$ [25]
Start point	“0” which is linked directly to only J1
Destination points	P1, P2, P3, P4, P5, and P6

**Table 3:** Set of preferences and weights for the parking and routing algorithm.

Case	Factors	Weighting Values	Description
CASE-1	$d_{i1},d_{i2},d_{i3},d_{i4},d_{i5}$	0.2,0.2,0.2,0.2,0.2	Equally emphasize on all factors
CASE-2	$d_{i1},d_{i2},d_{i3},d_{i4},d_{i5}$	1/3,1/3,0,1/3,0	Equally emphasize on congestion, waiting time and availability factors
CASE-3	$d_{i1},d_{i2},d_{i3},d_{i4},d_{i5}$	0.4,0.2,0,0.4,0	Equally emphasize on congestion and availability factors and less on waiting time at gates
